# Restoration of hand function with long-term paired associative stimulation after chronic incomplete tetraplegia: a case study

**DOI:** 10.1038/s41394-019-0225-5

**Published:** 2019-10-01

**Authors:** A. Rodionov, S. Savolainen, E. Kirveskari, J. P. Mäkelä, A. Shulga

**Affiliations:** 1 BioMag Laboratory, Helsinki University Hospital, Helsinki, Finland; 2 Validia Rehabilitation Center, Helsinki, Finland; 3 Clinical Neurophysiology, Helsinki University Hospital and University of Helsinki, Helsinki, Finland; 4 Clinical Neurosciences, Neurology, Helsinki University Hospital, Helsinki, Finland

**Keywords:** Medical research, Spinal cord injury, Motor control, Spine plasticity, Long-term potentiation

## Abstract

**Introduction:**

This case study explores the gains in hand function in an individual with a chronic spinal cord injury (SCI). The intervention was long-term paired associative simulation (PAS). We aimed to provide PAS until full recovery of hand muscle strength occurred, or until improvements ceased.

**Case presentation:**

A 46-year-old man with traumatic C7 AIS B tetraplegia was administered PAS three times per week. After 24 weeks, PAS was combined with concomitant motor training of the remaining weak hand muscles. Outcome measures included the manual muscle test (MMT), motor-evoked potentials (MEPs), F-responses, hand functional tests, and the spinal cord independence measure (SCIM).

**Discussion:**

After 47 weeks of PAS the subject had improved self-care and indoor mobility and was able to perform complex motor tasks (SCIM score improved from 40 to 56). His left hand regained maximum MMT score (total 75; increase of score from baseline condition 19); the effect remained stable in the 32-week follow up. In the right-hand muscles, MMT scores of 4–5 were observed in follow up (total 71; increase from baseline 48). Improved values were also observed in other outcomes. This is the first demonstration of long-term PAS restoring muscle strength corresponding to MMT scores of 4–5 in an individual with chronic SCI. The effect persisted for several months, indicating that PAS induces stable plastic changes in the corticospinal pathway.

## Introduction

Spontaneous or therapy-induced recovery after spinal cord injury (SCI) depends on the number of spared and restored neural connections. A large proportion of individuals with SCI retain residual connectivity [1]. This residual connectivity is the main target for numerous experimental approaches attempting to restore signal transmission in the corticospinal tract and gain motor control over paralyzed muscles [2–4].

In paired associative simulation (PAS), descending neuronal volleys induced by transcranial magnetic stimulation (TMS) of the motor cortex are timed to coincide at the cortical [5] or spinal level [6, 7] with antidromic volleys elicited by peripheral nerve electrical stimulation (PNS). Continuous pairing of pre- and postsynaptic inputs can change synaptic efficacy [8]. A number of in vivo studies suggest that pre-postsynaptic stimulation is important in reinforcing residual corticospinal connectivity and promoting recovery [9–11]. PAS can produce long-term potentiation (LTP)-like plasticity that appears immediately after PAS and persists for several hours in the corticospinal tract [7, 8]. These transitory neuroplastic changes occur in healthy people [8] and in individuals with stroke [12] and SCI [13, 14].

A novel PAS protocol with a high-frequency peripheral component [6, 15] produces a robust potentiation of motor-evoked potentials (MEPs). It restored some voluntary movements in several individuals with chronic traumatic and disease-induced para- and tetraplegia [16–18].

This case study explores the gains in hand function in an individual with a chronic SCI (SCI). The intervention was long-term PAS. We aimed to provide PAS until full recovery of hand muscle strength occurred, or until improvements ceased.

## Case presentation

A 46-year-old male with an incomplete tetraplegia (AIS B, neurological level C7), 5 years post injury consented to participate. The study was approved by the Ethics Committee of the Hospital District of Helsinki and Uusimaa. Conventional rehabilitation, including weekly physical therapy for 1–2 h, occupational therapy for 1 h, and pool therapy for 1–2 h, was maintained during the PAS and was the same as before the intervention. His muscle strength had remained stable prior to PAS (Supplementary Table 1A). Mean ± SD spontaneous changes of manual muscle test (MMT) score in the 17 months preceding PAS were 0.67 ± 0.52 (right hand) and 0.17 ± 1.17 (left hand). Pinch strength values were 0.57 ± 0.32 kg (right hand) and 0.27 ± 0.63 kg (left hand) in the 12 months preceding PAS (Supplementary Table 1B). Medication is presented in Supplementary Table 1C.

**Fig. 1:**
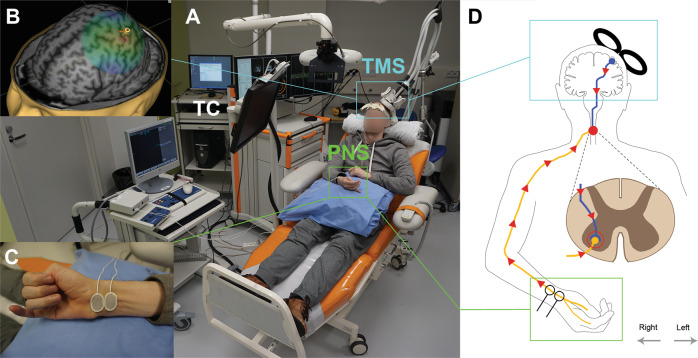
**a** Stimulation setup for long-term PAS therapy, **b**, **c** stimulation sites, and **d** basic principle of PAS. The upper motor neurons in the left primary motor cortex were stimulated with the TMS coil placed over the subject’s head (marked with blue squares). The electric field and a stimulated hotspot in the brain were visualized online using a 3D MRI-based model (**b**) that ensures accuracy and repeatability of stimulation. **c** High-frequency electrical stimulation was delivered to a peripheral nerve of the contralateral hand (marked with green squares). **d** The interstimulus interval was adjusted for collision of multiple descending volleys propagating along the upper motor neurons within the corticospinal tract (dark blue line) and ascending volleys travelling along the lower motor neurons within the peripheral nerve (yellow line) at the level of their synaptic contacts (red circle) in the cervical segment of the spinal cord (shown in enlarged form in **d**)

Navigated TMS (nTMS) of the motor cortex was delivered by a NBS 4.3 stimulator (Nexstim Ltd., Helsinki, Finland) with a figure-of-eight coil (outer diameter 70 mm, biphasic pulse). Structural T1-weighted MRI for nTMS was obtained with a 3T Siemens Verio scanner (Siemens Healthcare, Erlangen, Germany). The nTMS system enables accurate, reproducible stimulation [19] of selected cortical sites, the “hotspots” (Fig. 1a), where MEPs are most readily elicited from the selected muscles [17]. The hotspots were defined for abductor pollicis brevis (APB), abductor digiti minimi (ADM), and brachioradialis (BR) muscles in both hands as in our previous studies [17].

PNS was delivered using a Dantec Keypoint electroneuromyography device (Natus Medical Inc., Pleasanton, CA, USA) and surface electrodes (Fig. 1b). The nerves were stimulated as in our previous studies [17]. Initially, PNS stimuli were 50-Hz trains (1-ms square pulses, 6 pulses per train, train duration 100 ms). From week 30 onwards, 100-Hz trains (train duration 50 ms) were used as they were experimentally proven to be more efficient for MEP potentiation in healthy subjects [20].

For PAS (Fig. 1), nTMS (single-pulse, 0.2 Hz, 100% MSO) of the selected hotspots was synchronized with the first pulse of the PNS train given to the corresponding contralateral nerve [17]. The interstimulus interval for each pair was calculated using individual parameters (Table 1) [6]. The subject was seated comfortably during PAS. One session consisted of PAS of 3–6 hotspot-nerve pairs given in pseudo-random order of 1.5–3 h duration in total (20 min per nerve plus time for preparations, Fig. 1).

**Table 1:** Stimulation parameters and patient instructions

Side	Nerve	PNS		F (ms)	MEP (ms)	ISI (ms)	Instructions
		Weeks 1–13 (mA)	Weeks 14–47 (mA)				
Right	Median	6.6	6	30.9	34.0	−3	Pull thumb, index, and middle finger together before a TMS pulse and relax them after the pulse
	Ulnar	12.1	11	32.1	28.0	4	Bend IV–V fingers before the pulse and relax after the pulse (10 min), spread all fingers (10 min)
	Radial	16.5	12	20.3	16.9	3	Imagine opening the palm
Left	Median	11	10	20.3	24.7	−4	Imagine pulling thumb, index, and middle finger together
	Ulnar	14.3	13	30.6	27.8	3	Imagine bending IV–V fingers (10 min) or spreading all fingers (10 min)
	Radial	33	15	21.6	18.0	4	Imagine opening the palm

PNS, 110% of minimum stimulus intensity that produced visually distinguishable F-response; F, minimum F-wave latency; MEP, mean MEP latency. ISI was calculated by subtracting MEP from F (see [6] for details). If ISI is a positive value then in each stimulus pair PNS preceded TMS; if the value is negative, PNS and TMS pulses were given in opposite order. Right median and ulnar nerve stimulation were coupled with hand activation instead of imagery since RMT at initial motor cortex mapping was over 100%. PNS intensity for ulnar and median nerves in both hands was decreased by 10% after 13 weeks of stimulation. At the same time, PNS intensity for the right radial nerve was reduced to 12 mA and for left radial nerve to 15 mA according to new values obtained after new F-response measurement. These intensities were used for the remainder of stimulations. F latency and MEP latency values were obtained prior to the intervention. PAS of the right radial nerve and all stimulated nerves in the left hand was coupled with motor imagery. Right median and ulnar nerve PAS was coupled with muscle activation instead of imagery since RMT at initial motor mapping was over 100%

The hand motor training (MT) combined with the PAS (PAS-MT) was aimed at increasing muscle strength in those muscles where MMT scores remained 2 or less after 24 weeks of the intervention. Three weak muscles in the right hand were trained with motor tasks (thumb palmar abduction for APB; spreading fingers for dorsal interossei; and thumb radial abduction for abductor pollicis longus) simultaneously with the corresponding PAS. AR manually assisted the subject in finger movements.

**Fig. 2:**
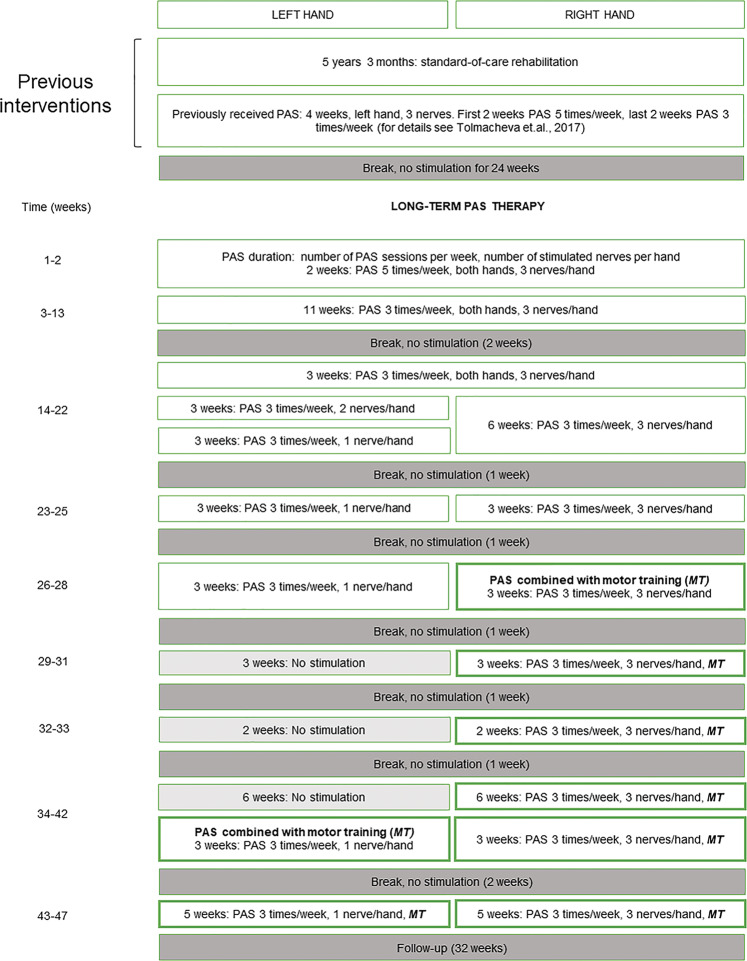
Time course of the intervention

The intervention of 56 weeks included 47 weeks of PAS and 1–2-week breaks without stimulations (Fig. 2). PAS to a hotspot-nerve pair was stopped when strength and range of motion of all muscles innervated by this nerve reached an MMT score of 4–5. All left-hand muscles reached this level at week 28; only the right hand was stimulated thereafter. At subject’s request, PAS of the left median nerve and the associated motor task were reinitiated at week 40 to enable more fluent grasp movements. The follow-up period started when the MMT scores of all evaluated muscles of both hands reached level 4 or 5 and was continued for 32 weeks. All tests were performed immediately prior to the study, during the intervention, and follow up.

Outcome measures in both hands included MMT (total scores for all evaluated muscles and partial scores of the muscles innervated by each of the stimulated nerves) [21] and the modified Ashworth scale (MAS, spasticity in wrist and elbow) evaluated by a physiotherapist specialized in SCI and blinded to the intervention changes and results of previous evaluations. In addition, we collected sensory scores of the American Spinal Injury Association impairment scale (AIS); grip strength assessed with the adjustable-handle Jamar dynamometer (Asimov Engineering Co., MA, USA); tip, key, and palmar pinch assessed with the pinch gauge (B&L Engineering, CA, USA); the spinal cord independence measure (SCIM); the box and block (BB) test; the nine hole peg test (NHPT); and MEPs and F-responses recorded from APB, ADM, and BR [17]. For the MMT, only muscles that scored <5 in the first assessment were selected for further evaluation (Supplementary Table 2 parts 1, 2). The subject reported subjective functional changes.

**Fig. 3:**
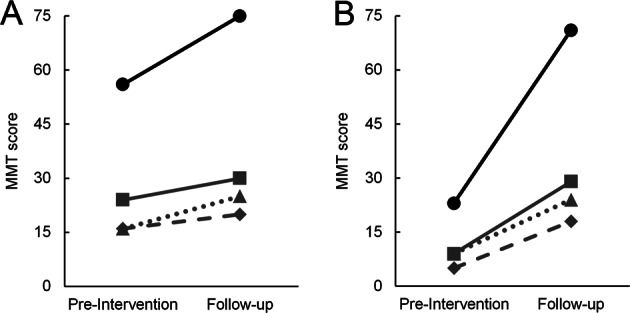
Manual muscle test (MMT) score measured before intervention, and at the end of follow up in the left (**a**) and right (**b**) hand (full MMT data is shown in Supplementary Table 2 parts 1, 2). *Y*-axis depicts the MMT score (the highest possible MMT score for all muscles evaluated is 75). Solid black line and spheres indicate the sum of MMT score calculated for all evaluated muscles. Solid grey line and squares indicate the sum of MMT score for the muscles innervated by median nerve, ulnar nerve values are indicated by dotted line and triangles, and radial nerve values by dashed line and diamonds (see Supplementary Table 2 parts 1, 2)

**Fig. 4:**
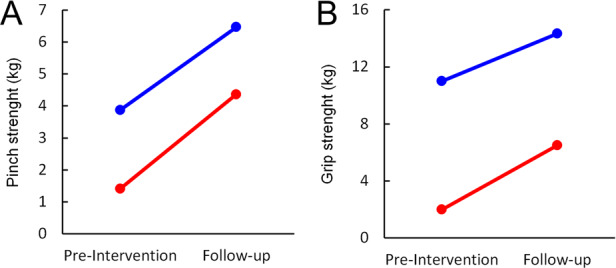
Changes in hand strength between pre-intervention and the end of the follow-up period. **a** The sum of tip, key, and palmar pinch tests results in the right (red) and left (blue) hand. **b** Grip tests in both hands (red, right hand; blue, left hand). *Y*-axis represents weight in kg. See Supplementary Table 4 for full pinch tests results

**Fig. 5:**
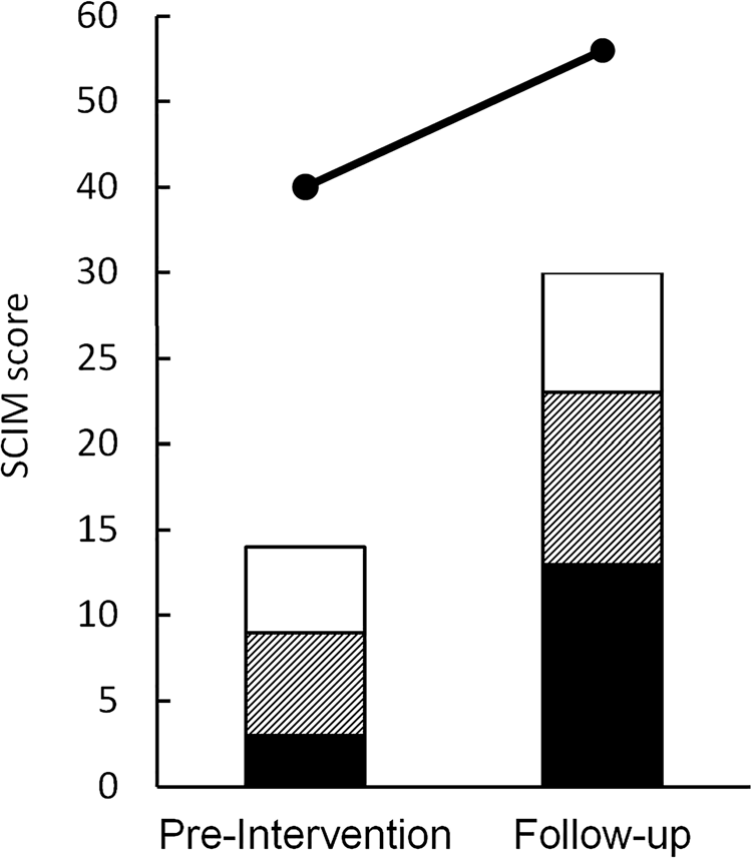
Spinal cord independence measure (SCIM) scores (*Y*-axis) before intervention and at the end of follow up. Black bars indicate self-care, bars with diagonal lines indicate mobility (room and toilet), white bars indicate mobility (indoors and outdoors) subscales, and black line depicts change in total SCIM scores. See Supplementary Table 6 for details

The total and partial MMT scores were improved in both hands (Fig. 3 and Supplementary Table 2 parts 1, 2). In total, the left hand regained 19 points and the right hand 48 points during the intervention. The scores reached the maximum level in the left hand after 47 weeks and remained stable in follow up. MMT scores of level 4 or 5 in the right-hand muscles were obtained in follow up. MMT scores were improved further during the PAS-MT, particularly in the right hand (Supplementary Table 2 part 1). AIS sensory scores did not differ (Supplementary Table 3). Spasticity assessed with MAS remained 0 at all times. Increased grip and pinch strengths were observed in both hands already after 8–20 weeks of PAS. Remarkably, all tested pinch strength types were increased (Supplementary Table 4). In the right hand, grip and pinch strength increased during the first 8–20 PAS weeks and stabilized thereafter. In the left hand, the increase in pinch strength stabilized after 25 weeks of PAS. Grip strength increased in the right hand by 4.5 kg and in the left hand by 3.3 kg (Fig. 4). Pinch strength increased in the right hand by 3.0 kg and in the left hand by 2.6 kg, (Supplementary Table 4). PAS-MT further increased pinch strength. Pinch strength decreased towards the level observed before PAS-MT administration in follow up. Results of the BB test increased from 45/68 (right hand/left hand) blocks to 52/75 blocks. By the end of the intervention, the NHPT time decreased by 31 s in the left and by 32 s in the right hand (Supplementary Table 5) and remained stable during follow up in the left hand (see Supplementary Video 1). The subject’s self-care and indoor and outdoor mobility increased during the PAS and improved further during follow up. His SCIM self-care score increased from 3 to 13 and SCIM indoor mobility from 6 to 10 (Fig. 5). Respiration and sphincter management did not change. Before PAS, the subject needed total or partial assistance in eating, bathing, dressing, and grooming. During follow up, he could perform these tasks independently and without adaptive devices. In total, the subject’s SCIM score increased from 40 to 56 (Supplementary Table 6). He enhanced his coherent motor control after 8 weeks of PAS (see Supplementary Videos 2 and 3), and reported numerous improvements in both hands during follow up (Table 2). He regained the ability to perform various complex fine motor tasks without external help. Before intervention, the subject had pain and uncomfortable tingling that decreased or completely disappeared during the intervention and follow-up (Supplementary Table 7).

**Table 2:** Motor improvements

Hand	Movement	Before	After
Right	Grasp	Only supporting function; could not be used independently	Can grasp objects and hold them in the hand in a stable manner
Right	Pressing buttons/switches	Unable to press different buttons and switches, e.g. toilet flush button	Able to press buttons/switches with ease
Left	Twirl	Could not twirl objects	Can twirl in the left hand
Both	Grasp, pinch	Could not do proper grasps with either right or left hand. Movement not coordinated; help needed in fine motor tasks	Can take books from the shelf and browse them, hold a paper sheet, and use different machines and devices (with small buttons or switches)
	Pressing buttons/switches, sliding	Could hold cell phone in the right hand and tap with the left hand	Can use cell phone and computer in a more versatile way with either the right or left hand
	Opening shirt buttons	Several minutes needed to unbutton a shirt; sometimes not possible altogether	Can open collar buttons in tens of seconds
	Wearing mittens	Could not put on mittens independently	Able to put on mittens
	Writing	Had to lift arms and elbows when typing due to finger weakness; strong pain in the neck and right shoulder and sometimes in the left shoulder associated with typing	The need to lift forearms gradually disappeared and was associated with ameliorated pain; typing speed increased and ability to work improved

**Fig. 6:**
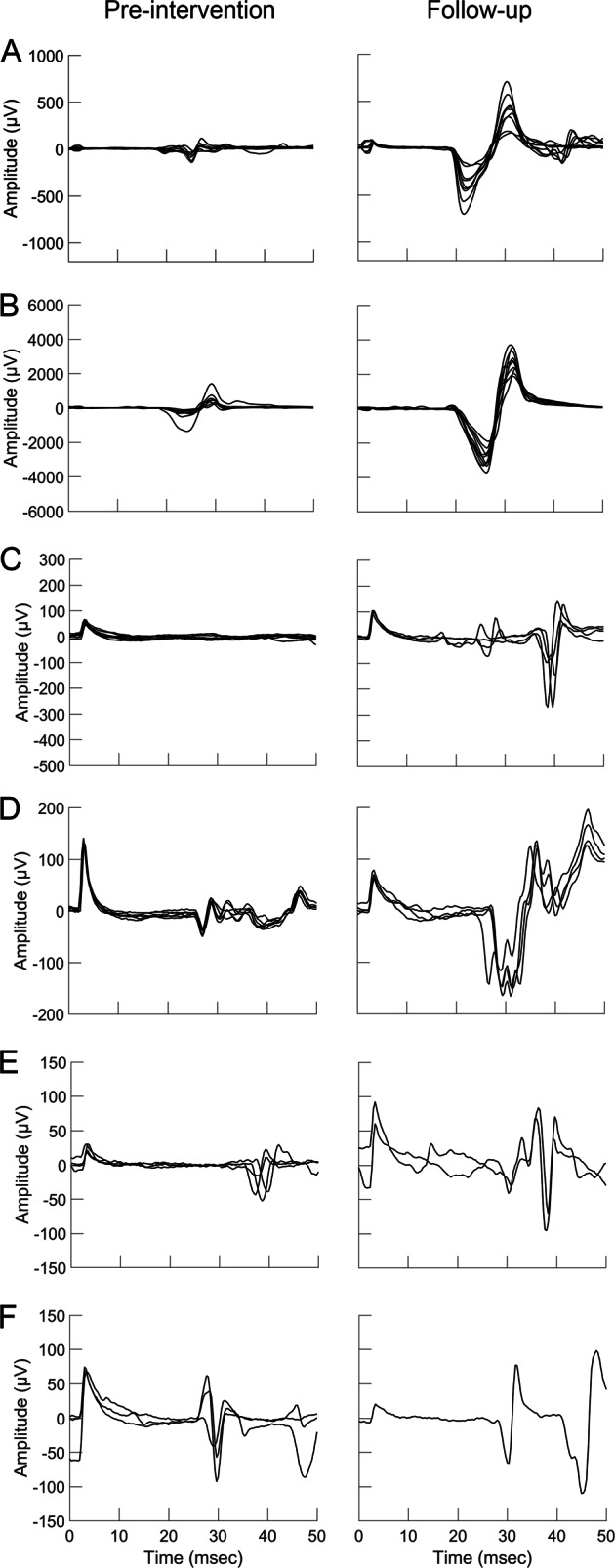
Representative motor-evoked potentials (MEPs) elicited by magnetic stimulation of the motor cortex hotspots used for PAS therapy. Responses from the right (**a**) and left brachioradialis (**b**), right (**c**) and left abductor digiti minimi (**d**), and right (**e**) and left abductor pollicis brevis (**f**) muscles recorded before the intervention and at the end of follow up. Each image consists of 1–10 superimposed MEPs. *X*-axis indicates time in ms (zero value corresponds to TMS onset; note the stimulus artefact). *Y*-axis indicates MEP amplitude in µV. Amplitude scales differ between responses from different muscles

The PAS potentiated MEPs in five out of six targeted muscles (Fig. 6). The overall effect differed slightly between the right and left hands. In all six muscles, the MEP amplitudes in both hands increased on average by 324% after 16 weeks of follow up when compared with the corresponding values obtained prior to PAS. Before treatment it was not possible to elicit MEPs from the right ADM even with muscle preactivation. MEPs were detected after 42 weeks of stimulation and remained stable during follow up. In the left ADM, small MEPs were elicited before PAS. Their amplitudes increased during PAS and follow up. Although the changes were variable, the total amplitude increase was systematic when the first and last MEP recordings were compared (Fig. 6). The minimum F-response latencies (F min) in the right hand decreased after PAS from 32 to 25 ms (23%) for the ulnar nerve and from 31 to 28 ms (9%) for the median nerve. No response was found with the left median nerve before intervention but was obtained during follow up (F min = 33 ms). F-min latency in the left ulnar nerve (31 ms) did not change. F-responses to the radial nerve stimulation in both hands were inconsistent. No amplitude changes were observed.

Around week 12, during PAS of the right radial nerve the subject reported a sensation in both legs resembling electrical stimulation, leg spasticity, and spasticity-related leg pain. A simultaneous urinary tract infection unrelated to stimulation was detected and treated. Psychological stress unrelated to stimulation occurred simultaneously. The PAS was interrupted for 2 weeks and the subject increased pain medication. After that he reported a decrease in spasticity and pain. The subject’s position in the chair was adjusted to increase comfort, and PNS intensity was slightly decreased (Table 1). During weeks 16–19 these symptoms gradually disappeared. No other adverse effects were reported.

## Discussion

Neuromodulation, in combination with intensive rehabilitation, is increasingly applied in human SCI research. Spared corticospinal connectivity can be utilized to restore function after SCI [4, 16–18, 22–26]. We show for the first time that a person with chronic tetraplegia can regain normal hand MMT scores by means of long-term PAS. The effects were clearly more profound than fluctuations of muscle strength observed prior to the intervention, excluding spontaneous recovery (Supplementary Table 1, Fig. 4). The findings are consistent with our previous studies [16–18], where shorter treatment periods were used. Here we reveal a more profound and persistent improvement that continued for several months after PAS.

We observed an increase in gross hand function and an enhanced performance of each individual muscle and notable improvements in SCIM. PAS plausibly strengthens residual connectivity in the hand motor pathways [6, 15]; the more versatile use of hands in daily life associated with this change further improves motor capability during and beyond the PAS period. As PAS affects the peripheral nerves, spinal cord, and motor cortex, it can modulate excitability in the descending pathways connected to the selected muscles even if natural signal transmission across these pathways is insufficient for eliciting movement. Activation of these precisely defined cortex–nerve pairs might be particularly beneficial in hand rehabilitation, where obtaining separated fine-finger movements is essential. Moreover, since incomplete SCI is often asymmetric and deficits can be unilateral, it may be clinically meaningful to focus only on the connections injured most extensively.

PAS does not require stimulation to be switched on during movement and hence does not interfere with proprioceptive pathways during motor rehabilitation. The peripheral component of PAS induces antidromic activation of lower motor neurons and also activates orthodromic pathways that mediate proprioceptive feedback. Recruitment of afferent fibres conveying proprioceptive information is thought to be imperative for neuromodulatory techniques, such as epidural and transspinal electrical stimulation, to increase the excitability of the residual spinal pathways. However, neuromodulation should avoid interfering with the natural proprioceptive feedback elicited by movements during motor rehabilitation [27].

In our previous study, the subject in this study had undergone a shorter intervention (Fig. 2) [16]. The positive results of this short PAS intervention were not comparable to the more extensive improvement from this longer PAS administration. Our study is limited to results of one subject only. A larger sample with a longer duration of the intervention would be required to demonstrate the efficacy of this intervention.

This study is the first to demonstrate that normal strength and range of movement of individual hand muscles can be recovered by means of long-term PAS in a subject with a chronic incomplete tetraplegia. We also show for the first time a sustained improvement in SCIM scores by PAS.

The TMS and PNS equipment is available in many hospitals and laboratories around the world. The measurements needed prior to PAS employ basic neurophysiological methods. Patients with less severe injuries plausibly need shorter stimulation times than our subject. Our PAS protocol does not require precise adjustment of the interval between TMS and PNS [15], or precise mapping of the cortex [20]; the protocol is thus feasible in clinical settings. PAS is non-invasive. Our results justify further investigation and development of long-term PAS protocols for the rehabilitation of individuals with SCI at chronic and subacute stages.

## Supplementary information

Supplementary Video 1

Supplementary Video 2

Supplementary Video 3

Supplementary Table 1A

Supplementary Table 1B

Supplementary Table 1C

Supplementary Table 2 first part

Supplementary Table 2 second part

Supplementary Table 3

Supplementary Table 4

Supplementary Table 5

Supplementary Table 6

Supplementary Table 7

## Data Availability

The data can be provided by the authors on request.

## References

[1] Ackery A, Tator C, Krassioukov A (2004). A global perspective on spinal cord injury epidemiology. J Neurotrauma.

[2] Cristante AF, Barros Filho TEP, de, Marcon RM, Letaif OB, Rocha IDda (2012). Therapeutic approaches for spinal cord injury. Clinics.

[3] Dietrich WD (2015). Protection and repair after spinal cord injury: accomplishments and future directions. Top Spinal Cord Inj Rehabil.

[4] James ND, McMahon SB, Field-Fote EC, Bradbury EJ (2018). Neuromodulation in the restoration of function after spinal cord injury. Lancet Neurol.

[5] Stefan K, Kunesch E, Cohen LG, Benecke R, Classen J (2000). Induction of plasticity in the human motor cortex by paired associative stimulation. Brain.

[6] Shulga A, Lioumis P, Kirveskari E, Savolainen S, Mäkelä JP, Ylinen A (2015). The use of F-response in defining interstimulus intervals appropriate for LTP-like plasticity induction in lower limb spinal paired associative stimulation. J Neurosci Methods.

[7] Taylor JL, Martin PG (2009). Voluntary motor output is altered by spike-timing-dependent changes in the human corticospinal pathway. J Neurosci.

[8] Carson RG, Kennedy NC (2013). Modulation of human corticospinal excitability by paired associative stimulation. Front Hum Neurosci.

[9] McPherson JG, Miller RR, Perlmutter SI (2015). Targeted, activity-dependent spinal stimulation produces long-lasting motor recovery in chronic cervical spinal cord injury. Proc Natl Acad Sci USA.

[10] Nishimura Y, Perlmutter SI, Eaton RW, Fetz EE (2013). Spike-timing-dependent plasticity in primate corticospinal connections induced during free behavior. Neuron.

[11] Ahmed Z (2013). Electrophysiological characterization of spino-sciatic and cortico-sciatic associative plasticity: modulation by trans-spinal direct current and effects on recovery after spinal cord injury in mice. J Neurosci.

[12] Uy J, Ridding MC, Hillier S, Thompson PD, Miles TS (2003). Does induction of plastic change in motor cortex improve leg function after stroke?. Neurology.

[13] Roy FD, Yang JF, Gorassini MA (2010). Afferent regulation of leg motor cortex excitability after incomplete spinal cord injury. J Neurophysiol.

[14] Bunday KL, Perez MA. Motor recovery after spinal cord injury enhanced by strengthening corticospinal synaptic transmission. Curr Biol. 2012;22:2355–61. 10.1016/j.cub.2012.10.046 PMC3742448 23200989

[15] Shulga A, Zubareva A, Lioumis P, Mäkelä JP (2016). Paired associative stimulation with high-frequency peripheral component leads to enhancement of corticospinal transmission at wide range of interstimulus intervals. Front Hum Neurosci.

[16] Tolmacheva A, Savolainen S, Kirveskari E, Lioumis P, Kuusela L, Brandstack N (2017). Long-term paired associative stimulation enhances motor output of the tetraplegic hand. J Neurotrauma.

[17] Shulga A, Lioumis P, Zubareva A, Brandstack N, Kuusela L, Kirveskari E (2016). Long-term paired associative stimulation can restore voluntary control over paralyzed muscles in incomplete chronic spinal cord injury patients. Spinal Cord Ser Cases.

[18] Tolmacheva A, Savolainen S, Kirveskari E, Brandstack N, Mäkelä JP, Shulga A. Paired associative stimulation improves hand function after nontraumatic spinal cord injury: a case series. Clin. Neurophysiol. Pract. 2019;4:178–83. 10.1016/j.cnp.2019.07.002 PMC6921158 31886442

[19] Lioumis P, Zhdanov A, Mäkelä N, Lehtinen H, Wilenius J, Neuvonen T (2012). A novel approach for documenting naming errors induced by navigated transcranial magnetic stimulation. J Neurosci Methods.

[20] Tolmacheva A, Mäkelä JP, Shulga A (2019). Increasing the frequency of peripheral component in paired associative stimulation strengthens its efficacy. Sci Rep.

[21] Hislop HJ, Avers D, Brown M, Daniels L Daniels and Worthingham’s muscle testing: techniques of manual examination and performance testing. Elsevier; 2014. p. 514.

[22] Harkema S, Gerasimenko Y, Hodes J, Burdick J, Angeli C, Chen Y (2011). Effect of epidural stimulation of the lumbosacral spinal cord on voluntary movement, standing, and assisted stepping after motor complete paraplegia: a case study. Lancet.

[23] Wagner FB, Mignardot J-B, Le Goff-Mignardot CG, Demesmaeker R, Komi S, Capogrosso M (2018). Targeted neurotechnology restores walking in humans with spinal cord injury. Nature.

[24] Rupp R, Gerner HJ (2007). Neuroprosthetics of the upper extremity–clinical application in spinal cord injury and challenges for the future. Acta Neurochir Suppl.

[25] Freyvert Y, Yong NA, Morikawa E, Zdunowski S, Sarino ME, Gerasimenko Y (2018). Engaging cervical spinal circuitry with non-invasive spinal stimulation and buspirone to restore hand function in chronic motor complete patients. Sci Rep.

[26] Gad P, Lee S, Terrafranca N, Zhong H, Turner A, Gerasimenko Y (2018). Non-invasive activation of cervical spinal networks after severe paralysis. J Neurotrauma.

[27] Formento E, Minassian K, Wagner F, Mignardot JB, Le Goff-Mignardot CG, Rowald A (2018). Electrical spinal cord stimulation must preserve proprioception to enable locomotion in humans with spinal cord injury. Nat Neurosci.

